# Lamins’ role in osteosarcoma

**DOI:** 10.3389/fcell.2026.1783487

**Published:** 2026-03-02

**Authors:** Giulia Bagnato, Barbara Peruzzi

**Affiliations:** 1 DAHFMO – Unit of Histology and Medical Embryology, Sapienza University of Rome, Rome, Italy; 2 Department of Life Science and Public Health, Section of Histology and Embryology, Catholic University of Sacred Heart, Rome, Italy; 3 Oncology and Experimental Neuroscience Unit, IRCCS Fondazione Policlinico “Agostino Gemelli”, Rome, Italy

**Keywords:** bone tissue, lamins, mechanotransduction, nuclear envelope, osteosarcoma

## Abstract

Osteosarcoma (OS) is a rare and highly aggressive bone tumor that can develop in several skeletal segments, although it predominantly affects the long bones. This cancer mostly occurs in adolescents, young adults and people older than 60. There are many questions still open regarding osteosarcoma biology and the efficacy of current treatments. Recent research has increasingly emphasized the critical role of mechanotransduction as a key regulator of cellular functions. Notably, emerging evidence highlights the nucleus as an active player in mechanosensing and mechanotransduction processes within the bone tissue. The nuclear envelope is composed of several proteins, among which lamins. These proteins are essential components of the inner nuclear membrane (INM) exerting many different functions, also known for having a pivotal role in mechanotransduction and bone cell differentiation. In this review, we analyze the state of the art regarding the lamins role in bone cancer biology.

## 1 Introduction

Osteosarcoma (OS) is a relatively uncommon type of cancer, accounting for approximately 1% of all cancer diagnoses worldwide. This malignancy is frequently observed in teenagers and young adults (Liu et al., 2025). OS is a highly aggressive bone tumour that can develop in different skeletal locations but predominantly appears in the long bones, particularly affecting regions around the knee joint (Ritter and Bielack, 2010). OS originates from mutated mesenchymal stromal cells (MSCs), leading to the formation of malignant osteoblasts and the deposition of abnormal and aberrant bone matrix. The proliferation rate of malignant osteoblasts is higher than normal osteoblasts. Despite the recent advances review by Brar et al., many questions regarding osteosarcoma and the efficacy of therapies remain unanswered (Brar et al., 2025). In recent years, different studies have pointed out the importance of mechanotransduction as a regulating factor for development, migration, differentiation, and other cell functions involving the activation of integrin- and cytoskeleton-depending pathways (Tschumperlin et al., 2011). Indeed, the external mechanical information needs to be received by the nucleus to modulate and respond to the stimulus. In addition, many studies have pointed out that the deregulation of mechanotransduction plays a role in cancer at different levels and have assumed that the study of mechanotransduction pathways could lead to the development of new therapeutic strategies, even in osteosarcoma (Corre et al., 2020; Luu and Viloria-Petit, 2020; Shoaib et al., 2022). Although the clear evidence about mechanotransduction involvement in cancer, there are limited data about the nuclear envelope and its role in the mechanobiology of bone cancer. In this context, the nucleus was considered a passive organelle for a long time, designated only to protect DNA. Recent findings have highlighted its relevance and active participation in mechanosensing and mechanosignaling processes in bones (Birks and Uzer, 2021). The nuclear envelope is constituted by many different proteins and is connected to the cytoplasm through the LINC complex, a very important structure that not only provides a connection between the two compartments but is also involved in maintaining cell mechanosensitivity (Alam et al., 2016). We suggest to consider the involvement of nuclear envelope in the mechanobiology of osteosarcoma, a field of growing interest. This review focuses on lamins, key proteins of the inner nuclear membrane (INM) that have long been recognized for their crucial role in mechanotransduction (Swift et al., 2013).

## 2 Lamins

Lamins are intermediate filaments that provide structure to the nucleus and participate in many other functions related to chromatin organization, gene transcription and epigenetic modifications (Bianchi et al., 2020; Dhankhar et al., 2025; Dechat et al., 2008). A-type lamins (Lamin-A and Lamin-C) are encoded by alternative splicing of the *LMNA* gene, and their expression in cells is related mainly to the differentiation state and tissue stiffness (Swift et al., 2013). B-type lamins are encoded by the *LMNB* gene and are crucial for development during embryogenesis (Evangelisti et al., 2022). Nmezi et al. demonstrated that Lamin A/C and Lamin B are distributed differently: Lamin A/C forms a tighter meshwork, while Lamin B is closer to the INM but in a less dense manner. It has been shown that cells that express more Lamin A/C have greater nuclear rigidity and consequently follow the deformation less (Nmezi et al., 2019). Low Lamin A/C values allow greater nuclear deformation and consequently an increase in migratory capacities, unlike high Lamin A/C values, in which cell migration seems reduced (Harada et al., 2014). Lamin A/C contributes to the mechanical stiffness of the nucleus, while Lamin B stabilizes the nuclear membrane structure, supplementing the role of Lamin A/C (Lammerding et al., 2006). It is worth noting that Lamin A/C is the last mechanotransductor, connecting the nucleus with the cytoskeleton through the LINC complex, and this connection is essential for nuclear mechanotransduction and its mechanical properties (Lee et al., 2023). There is a lot of evidence proving the involvement of lamins in nuclear mechanics and mechanostraduction. For example, the absence of Lamin A/C in fibroblasts is marked by compromised nuclear mechanics and mechanotransduction under mechanical stress. This is evidenced by heightened nuclear deformations and increased nuclear fragility, diminished expression of mechanosensitive genes, and disrupted transcriptional activation, leading to reduced viability of cells subjected to mechanical strain (Lammerding et al., 2004; Lammerding and Lee, 2005). Moreover, chromatin is organized on the internal nuclear framework created by lamins, allowing external mechanical signals to modulate heterochromatin via LINC and Lamin A/C mechanotransduction (Evangelisti et al., 2020). Additionally, during development, Lamin A/C levels increase to provide mechanical protection to the genome. As LaminA/C is crucial for mechanotransduction, the mechanical strain experienced by Mesenchymal Stromal Cells (MSCs) affects their lineage differentiation acting on Lamin A/C expression. Matrix stiffness can determine the fate of MSCs, as the shape of the nucleus varies according to the substrate elasticity surrounding the cell. On softer substrates, the nuclear envelope appears wrinkled and relaxed, whereas on stiffer substrates, the nucleus is compressed by stress fibres and appears tense and smooth (Engler et al., 2006). Therefore, nuclear remodelling, depending on the substrate elasticity, could have important regulatory implications for cell differentiation and functionality. In this manner, the levels of Lamin A/C correlate closely with matrix elasticity: Lamin A/C levels increase with increasing tissue stiffness and decrease on softer matrices. Moreover, the relationship between Lamin A/C levels and matrix stiffness influences MSCs’ differentiation (Swift et al., 2013). Given that Lamin A/C levels correspond to both nuclear and tissue stiffness, researchers have largely focused on Lamin A/C as the primary mechanical component. Furthermore, Lamin A/C depletion resulted in only a roughly 50% reduction in nuclear stiffness, suggesting that other nuclear mechanical elements are responsible for the remaining structural integrity (Lammerding et al., 2004; Stephens et al., 2017).

### 2.1 Lamin A/C’s role in bone tissue

**FIGURE 1:**
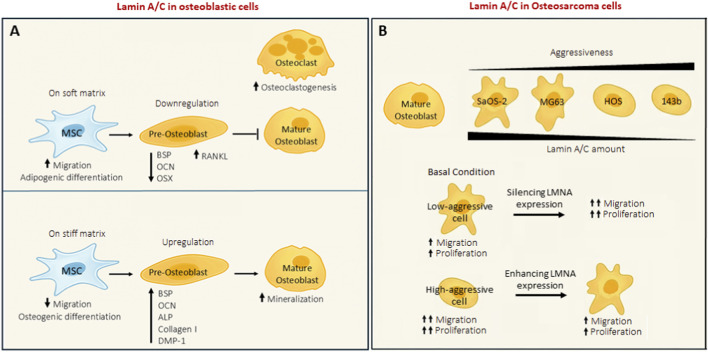
**(A)** Lamin A/C role in osteoblastic cells. **(B)** Lamin A/C modulation in OS cell lines with increasing aggressiveness.

In the context of bone tissue, lamins play a crucial role in bone differentiation and development (Alcorta-Sevillano et al., 2020). In particular, Lamin A/C is essential at different levels during MSCs’ commitment and during the maturation of osteoblasts. MSCs navigate towards stiffer regions of substrates, transitioning from a soft matrix (1 kPa) to a rigid matrix (34 kPa), by orienting the cytoskeleton (Raab et al., 2012). Given that Lamin A/C is intricately associated with the cytoskeleton through the LINC complex, it plays a role in cellular responses to matrix elasticity. Recent research indicates that physical and mechanical cues from the microenvironment are pivotal in determining MSC fate, suggesting a close relationship between the mechanical cues and the specific lineage differentiation of MSCs (Vahabikashi et al., 2022). Indeed, the nucleus serves as the primary regulator of cell fate, as it governs gene expression and its ability to react to external signals (Rubin et al., 2022). LaminA/C deficiency in MSCs and mature osteoblasts causes a dramatic reduction in the expression of specific bone cell markers, such as genes involved in mineralization and alkaline phosphatase expression, and of bone differentiation master genes (as OCN, OSX, and BSP) (Akter et al., 2009). Furthermore, the absence of Lamin A/C has important side effects on both osteoblasts and osteoclasts. In fact, osteoblasts are less differentiated and, as a consequence, osteoclastogenesis is enhanced (Rauner et al., 2009). Indeed, Lamin A/C influences the regulation of two proteins that control osteoclastogenesis: receptor activator of nuclear factor κ-B ligand (RANKL) and osteoprotegerin (OPG) (Akter et al., 2009). RANKL is produced by immature osteoblasts; therefore, when Lamin A/C is inhibited, osteoblast differentiation is arrested, resulting in an elevated level of RANKL, disrupting the balance of the RANKL/OPG ratio and fostering an osteoclastogenic environment. In contrast, Lamin A/C overexpression in mouse pre-osteoblastic MC3T3-E1 cells has been demonstrated to enhances osteoblast differentiation and mineralization by stimulating the expression of alkaline phosphatase, type I collagen, BSP, OCN, and dentin matrix acidic phosphoprotein 1 (DMP-1) in the presence of bone morphogenetic protein 2 (BMP-2) growth factor (Tsukune et al., 2017). The main involvement of Lamin A/C in bone tissue is shown in Figure 1A. Moreover, Lamin A/C and LINC complexes are also responsible for the nuclear entry of β-catenin, YAP/TAZ, and Notch signalling, all of which play pivotal roles in osteoblast differentiation and maturation (Bermeo et al., 2015; Uzer et al., 2018; Swift et al., 2013). These evidences together pointout the importance of Lamin A/C in the maintenance of bone tissue homeostasis. Given the critical role of lamins in nuclear architecture and cellular function, mutations or dysregulation of these proteins can result in pathological conditions. Disorders arising from mutations in lamin genes are collectively referred to as laminopathies. The most studied one is HGPS, characterized by the accumulation of an immature form of Lamin A/C (pre-Lamin A/C) within the nucleus. This disease primarily affects bone and muscle (Eriksson et al., 2003). Beyond laminopathies, lamins can also be dysregulated in tumours, and in recent years there has been growing interest and research into their role in cancer.

### 2.2 Lamins’ involvement in cancers

The role of lamins in cancer is highly heterogeneous and closely depends on the specific type of cancer being examined. Experimental evidence of lamin deregulation has been found in several tumours, such as gastric, lung and pelvis carcinomas, resumed in Table 1. In lung adenocarcinoma, LMNB1 silencing inhibited cancer cell proliferation and migration (Tang et al., 2021). Anyway, the role of LMNB1 in lung cancer is controversial as other studies consider it as an onco-suppressor (Jia et al., 2019). While low levels of LMNA in lung cancer are associated with a poorer prognosis and more metastasis to bone (Kaspi et al., 2017). Moreover, a similar behaviour seems to be applied also in colorectal cancer by LMNB2 regulating p21 and promoting the cancer progression (Dong et al., 2021), also LMNA is considered a risk biomarker in colorectal cancer (Willis et al., 2008). There is contrasting evidence regarding colon cancer where the lower levels of LMNA expression were demonstrated to be related to a poorer prognosis (Belt et al., 2011). In hepato-cellular carcinoma (HCC), lamin family genes showed elevated expression levels. Prognostic evaluations indicated that LMNB1 and LMNB2 serve as indicators for poor clinical outcomes (Yang et al., 2022). It was demonstrated that combined targeting of LMNB2 alongside with a drug inhibition of PD1 exhibited a synergistic impact on hindering tumour advancement both *in vivo* and *in vitro*, especially in HCC models characterized by LMNB2 overexpression (Li et al., 2025). Regarding LMNA in HCC, this is considered an oncogene as its knockout cause a reduction in proliferation and migration capability of HepG2 cells (Liu et al., 2020). Interestingly, in prostate cancer the overexpression of LMNA is associated to a worst prognosis. In fact, has been demonstrated a correlation between the expression of LMNA in aggressive cancers and the invasiveness of tumours through the PI3K/AKT/PTEN pathway, suggesting that targeting LMNA could represent a chance to counteract the invasiveness of cancer (Kong et al., 2012). Also, LMNB1 is associated with tumour progression and metastasis in prostate cancer (Luo et al., 2021). Thereafter, even in breast cancer lamins deregulation was found. In particular, was demonstrated that highly invasive and rapidly proliferating breast cancer cells often display reduced levels of LMNA, which results in increased nuclear deformability, and as said before, this corresponds to an increased cell migration. Notably, elevating LaminA/C levels in highly invasive breast cancer cells specifically impact pathways associated with cell-ECM interactions and cellular metabolism (Bell et al., 2022; Setijono et al., 2018). Lastly, in ovarian cancer (OC) it is reported that higher levels of LMNB1 are associated with the advancement of the disease. Reducing LMNB1 expression slows down the growth and migration of OC cells. Moreover, in ovarian cancer LMNB1 is considered as a new potential prognostic marker and therapeutic target (Dong et al., 2023). Regarding LMNA in ovarian cancer, is known that its downregulation represents the trigger for nuclear abnormalities (Capo-chichi et al., 2011). Deeply, recent finding suggest that LMNA contributes to metastasis via nuclear morphologic changes (Ouchi et al., 2023).

**TABLE 1:** Involvement of Lamins in cancer.

Cancer type	A-type lamin involvement	B-type lamin involvement	Functional impact
Breast cancer (Setijono et al., 2018; Bell et al., 2022)	Downregulated	Upregulated	LMNB1 high is linked to aggressive disease and worse survival. LMNA low correlates with tumor progression
Colorectal cancer (Dong et al., 2021)	Upregulated/Loss	Upregulated	LMNB2 promotes cancer progression. Contrasting evidence regarding LMNA.
Hepatocarcinoma(Liu et al., 2020; Yang et al., 2022; Li et al., 2025)	Upregulated	Upregulated	LMNB1 promotes HCC progression. Knocking out the LMNA reduces cancer aggressiveness
Lung cancer (Jia et al., 2019; Tang et al., 2021)	Downregulated	Upregulated	Regarding LMNB1, evidence shows two different behaviors. Low levels of LMNA are associated with poorer prognosis
Osteosarcoma (Evangelisti et al., 2020; Fanelli et al., 2020; Urciuoli et al., 2020 ; 2021; Chiarini et al., 2022)	Downregulated	Upregulated	LMNA expression inversely correlates with tumor aggressiveness. LMNB2 modulates NER in DNA damage response
Ovarian cancer (Dong et al., 2023; Ouchi et al., 2023)	Upregulated	Upregulated	Higher levels of LMNB1 are associated with cancer progression. LMNA is upregulated is metastatic cancer
Prostate cancer (Kong et al., 2012; Luo et al., 2021)	Upregulated	Upregulated	LMNB1 is related to cancer progression. LMNA expression in aggressive cancers correlate with invasiveness

### 2.3 Lamins in bone cancer

At present, there are still few studies that have investigated the role of lamins in osteosarcoma but, given their relevance in the bone homeostasis, this could be key-pivotal point to be deepened in this field. Firstly, Urciuoli and co-authors demonstrated that osteosarcoma cell lines show a different lamin’s expression in comparison to normal osteoblasts. More in detail, Lamin A/C expression seems to be inversely related to the aggressiveness of tumour cell line, indeed the more metastatic ones have less content of Lamin A/C, while the Lamin B expression is always upregulated. The forced expression of Lamin A/C in high aggressive cell line reduces tumour features, such as proliferation and migration, and restores cell adhesion on substrate with bone tissue elasticity, meanwhile LaminA/C silencing in SaOS-2 (cells with low grade of aggressiveness) increases the proliferation rate (Urciuoli et al., 2021). Moreover, a correlation was also demonstrated between the expression of Lamin A/C and Emerin, YAP and Rb nuclear content (Urciuoli et al., 2020). In fact, their nuclear localization was reduced in A-type lamin deficient cells. As described before the nuclear content of YAP is regulated by A-type lamin and, as widely demonstrated, YAP is a mechanosensor protein important not only for the correct development of bone tissue (Dupont et al., 2011). These results suggest that Lamin A/C could play a role in OS progression, by then another study by Evangelisti et al., confirmed that elevated levels of Lamin A/C are directly involved in the migration and proliferation of OS cells (Evangelisti et al., 2020). There is another evidence in literature about the osteosarcoma and Lamin A/C expression. In this paper, the interaction between CNOT1 (transcription complex) in osteosarcoma cells was examined. They demonstrated that CNOT1 interacts with Lamin A/C and affects its stability, in fact, when CNOT1 is knockdown the levels of Lamin A/C decrease. The restoration of Lamin A/C protein expression in CNOT1-knockdown cells promoted tumour cell growth. The silencing of CNOT1 reduce osteosarcoma cell proliferation by blocking the Hedgehog signalling pathway through Lamin A/C. They suggest that strategies aiming at the CNOT1– Lamin A/C–Hedgehog signalling pathway axis may represent a practical and effective approach for osteosarcoma treatment (Cheng et al., 2017). This data gives to Lamin A/C a different role in osteosarcoma, which goes in contrast with the other available data. The main known function of Lamin A/C in OS are described in Figure 1B. Regarding the Lamin B1 in osteosarcoma has been demonstrated to modulate nucleotide excision repair (NER) in response to ultraviolet damage by adjusting the expression of genes related to NER (Butin-Israeli et al., 2013). Recent studies indicate that OS cells exploit DNA repair systems, such as NER, to evade DNA-damaging chemotherapeutics treatments, like cisplatin. A promising treatment approach may involve targeting NER components (Hattinger et al., 2020). Fanelli and co-authors recently discovered that two compounds, NSC130813 (NERI02; F06) and triptolide, which inhibit DNA repair by binding to DNA repair proteins, increase sensitivity to cisplatin in U2OS and SaOS-2 cells (Fanelli et al., 2020). Gaining insights into how these elements operate within the framework of mechanotransduction could enhance the efficacy of NSC130813 (NERI02; F06) and triptolide in treating resistant OSA patients. Another study conducted on Ewing’s sarcoma, which is another bone tumour, investigated the role of Lamin A/C in the metastatic process of Ewing’s sarcoma (EWS). They demonstrated that Lamin A/C expression is inversely correlated with tumour invasiveness in EWS patients. Into deep, Lamin A/C expression was significantly lower in metastatic tissues compared to primary tumours, and reduced Lamin A/C expression was associated with increased mobility and invasiveness of EWS cells. Furthermore, overexpression of Lamin A/C decreased motility and invasiveness of EWS cells *in vitro* and metastatic burden *in vivo*, particularly in liver metastases. This effect may be mediated by liver growth factors such as IGF and HGF/SF. The drug mevinolin, which increases pre-Lamin A/C levels, showed similar effects by reducing EWS cell migration and inducing neural differentiation, suggesting that Lamin A/C may promote differentiation in EWS cells. The study also highlighted the role of Lamin A/C in influencing the LINC complex, which regulates cytoskeleton dynamics and cell migration. Treatment with mevinolin reduced YAP/TAZ signaling activity, which is linked to tumor progression, and led to Lamin A/C accumulation. This treatment also reduced cell motility. In summary, the study identifies Lamin A/C’s role in the metastatic process of EWS and suggests that drugs like mevinolin, which modulate Lamin A/C, could be potential therapies for metastatic EWS cases (Chiarini et al., 2022).

## 3 Discussion

The mechanoenvironment plays a pivotal role in the pathophysiology of many different diseases and in quite recent years has been considered also in osteosarcoma, influencing tumour growth, metastasis, and resistance to therapy. Future research should focus on unravelling the complex interactions between mechanical forces and cellular responses in the OS microenvironment. Targeting the mechanoenvironment holds promise for developing novel therapeutic approaches to improve outcomes for patients with osteosarcoma (Luu and Viloria-Petit, 2020). In addition to the extensively researched mechanotransducers such as integrins, focal adhesion components, and downstream signalling molecules, it is crucial to consider also the nucleoskeletal elements as potential therapeutic targets. As said in the first part of review, the nucleus participates in mechanosensing and mechanotransduction as a highly dynamic entity capable of transmitting force via various molecules and molecular assemblies, including lamins, nuclear actin, and the Linker of Nucleoskeleton and Cytoskeleton (LINC) complex (Lambert, 2019). Furthermore, these nuclear proteins participate in cellular mechanisms that significantly impact cancer development, including DNA repair, transcription, and replication. As previously discussed, lamins play a crucial role in bone development and metabolism, particularly since they also control the nuclear entry of important transcription factors, such as β-catenin and YAP (Bermeo et al., 2015; Uzer et al., 2018; Swift et al., 2013). In recent years, several drugs that inhibit the Hippo pathway have been studied in osteosarcoma (Luu and Viloria-Petit, 2020). The results are promising but still require further investigation. Moreover, other studies have reported that inhibiting the WNT/β-catenin pathway is effective in reducing the osteosarcoma lung metastasis (Wang et al., 2022). Since Lamin A/C and the LINC complex are partly responsible for the nuclear entry of YAP and other deregulated proteins, and because lamins are known to be altered in cancer and contribute to the hallmarks of cancer (Dubik and Mai, 2020), it would be valuable to further investigate the role of Lamin A/C and other nuclear envelope proteins in osteosarcoma.

Finally, a hallmark of osteosarcoma is chromosomal instability (Espejo Valle-Inclan et al., 2025), resulting in a highly complex and unstable karyotype and extreme aneuploidy (Katsianou et al., 2025). This may be due, at least in part, to alteration in lamins expression and function in osteosarcoma cells, suggesting that also the epigenetic control could be a valuable tool to better characterize the cancer biology and to identify new potential adjuvant therapeutic targets (Lopez-Fuentes et al., 2025).
